# A DPSIR-TODIM Model Security Evaluation of China’s Rare Earth Resources

**DOI:** 10.3390/ijerph17197179

**Published:** 2020-09-30

**Authors:** Yupei Du, Wenju Wang, Qian Lu, Ziyang Li

**Affiliations:** 1 School of Aeronautics and Astronautics, Sichuan University, Chengdu 610065, China; duyupei123@gmail.com; 2 Business School, Sichuan University, Chengdu 610065, China; wangwenju@stu.scu.edu.cn (W.W.); 2018225025133@stu.scu.edu.cn (Q.L.); 3 Accounting Department, Sichuan University, Chengdu 610065, China

**Keywords:** rare earth elements, security evaluation, DPSIR, TODIM

## Abstract

Rare earth is an important strategic mineral resource for national economy and national security. As the largest producer and exporter of rare earth, China’s rare earth industry has problems associated with excessive production, mismatched pricing power and environmental pollution. Therefore, an in-depth study of the rare earth industry security is necessary. Based on proposed definition for mineral resource security, this paper established a rare earth resource security evaluation model based on the “driver-pressure-state-impact-response” conceptual model using an extended TODIM (an acronym in Portuguese for interactive and multi-criteria decision-making) method combined with the E-DEMATEL (entropy and decision-making trial and evaluation laboratory) method. The model was then applied to Chinese rare earth data from 2006–2015 to assess the security, from which it was found that while the security level was not high, the overall trend was improving. Moreover, some critical response factors affecting REEs (rare earth elements) security are identified, including tariffs, research investment, etc. This paper not only introduces a new evaluation of REEs security but also explores the crucial indicators and the response mechanism.

## 1. Introduction

### 1.1. Background

Rare earth elements (REEs) are widely used in new energy, new materials, energy conservation, environmental protection, aerospace, electronics, and other fields [1,2,3], and also have a strategic role in military applications such as guidance and control, targeting and weapon systems, and communication platforms [4]. Given the importance of REEs, it has been claimed that REEs shortages could be commercially [4,5] and strategically serious for many countries [6,7]. China has approximately 23% of the world’s total rare earth reserves and almost monopolizes the supply of REEs minerals, concentrates, and metals [8,9], because it has been satisfying 90% of the world’s demand for several decades [8]. Even so, China still cannot control the price of REEs [10]. Further, problems associated with China’s excessive REEs production, disordered REEs export orders, and the environmental pollution from REEs mining have severely limited rare earth industry development [11]. Although China strengthened its rare earth resource development and trade management at the beginning of the 21st century, the long-term disorderly development and utilization of the rare earth resources has resulted in an unprecedented rare earth security crisis in China [9]. Therefore, it is necessary to establish a security warning mechanism to promote the healthy and sustainable development of the rare earth industry.

### 1.2. REEs Resource Security

Mineral resource security has been examined in relation to energy security [12], so research on energy security could be consulted as a reference for understanding REEs resource security. Table 1 shows the evolution of a definition for energy/mineral resource security, from which it can be seen that energy/mineral resource security was initially defined from a market and economics perspective. With the growing emphasis on environmental damage and sustainable development during resource mining, research has now begun to examine energy/mineral resource security in terms of ecological, technological, and management risks. Generally, energy/mineral resource security research has focused on four main aspects: economic security (supply security, price stability), social security (production security, use security), ecological security, and international security (geopolitics, transportation road security, military security, etc.) The above scholars made analysis and evaluation of the overall situation of mineral resources, but in terms of rare earth as an important strategic mineral resource, what are the peculiarities compared to other resources? The uneven distribution and difficulty to substitute rare earths led to studies on their supply security and Chinese policy. Zhang et al. [13] exclusively dealt with supply security and criticality of Yttrium and found that Yttrium production causes a variety of environmental/sustainability issues, making the supply vulnerable to more stringent environmental policies. Reference [14] and Wübbeke [10] discussed China’s rare earth policy and proposed some policy implications, such as boosting innovation capability and establishment of a strategic resource reserve system. The current research on the security of rare earth resources has not formed a complete research system due to the different national interests represented by scholars and the relatively scattered research perspectives. This article takes the definition of resource security as the theoretical basis for developing new systematic and sustainable security assessment methods, explores the operating mechanism of rare earth security, and builds a theoretical framework of rare earth security. On the one hand, this issue is conducive to the enrichment and development of resource security and sustainable development. Secondly, it provides a reference for the state to manage superior strategic mineral resources.

### 1.3. Index Construction of Resource Security

To present rare earth resource security and system operation status scientifically and reasonably, the indicator analyses have been the most popular energy/resource security evaluation methods [23,24,25,26,27,28]. There have been three main security indicator system construction research directions, the first of which is index systems based on an energy security definitions [29,30,31,32,33] that integrate a quantitative dimension (resource endowment, supply and demand status), a qualitative dimension (technological development) and a space-time dimension (sustainability). The second direction has been based on the 4A index proposed by the Asia Pacific Energy Research Center [17], which suggests that energy supply security assessments should include: (1) physical energy security, that is, the availability and accessibility of supply sources; (2) economic energy security, that is, the affordability of resource acquisition and economic stability regardless of energy supply disruptions or high energy price shocks; and (3) environment sustainability. Research based on this direction has also added environmental, social, scientific, technological, and national security [34,35,36] dimensions. The third direction has been to adopt a PSR (pressure–state–response) model to construct the index system [37,38]. The PSR model was proposed by the International Organization for Economic Cooperation and Development (OECD) as a framework for studying environmental issues [39] and has also been used to evaluate petroleum, coal, and copper security [40,41]. While the above methods provide various ways to evaluate energy security, there have been two main problems with the construction of energy security index systems. First, there has been no standard definition offered for mineral resource security [23,42,43,44], and in some cases, the selected indicators have had strong correlations or logic mistakes, which have resulted in low indicator accuracy. Second, the system dimensions have been too small to give a full picture of the mineral resource security status or the vertical indicator changes. Therefore, it is urgent to construct a more comprehensive, accurate and effective evaluation system.

### 1.4. DPSIR Framework

**Figure 1:**
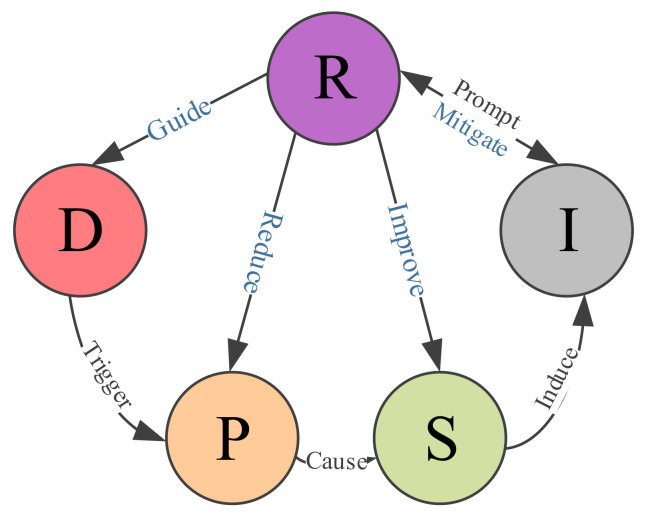
Driver Pressure–State–Impact-Response (DPSIR) model Schematic diagram.

Based on the PSR and DSR (driving force–state–response) model, the Driver Pressure–State–Impact-Response (DPSIR) model was proposed in 1993 by the European Environment Agency (EEA), and was subsequently used in environmental resource management and sustainable development evaluations [45], indicating the strength of the model. Fassio et al. [46] built a multi-criteria decision support system (MDSS) based on the DPSIR model and based on the comprehensive decision theory, and used it to simulate the impact of alternative policies on water resources. Then, it has been used for the security assessment of oil resources [47] and enables a comprehensive monitoring of the continuous feedback mechanism between the indicators, which shows the completeness and effectiveness of the DPSIR model. The model examines the interrelationship between people and the environment from a comprehensive perspective. Importantly, one of the main advantages of the DPSIR model is that it has strong logic and systematic content [48] and can identify the causal chain between the mineral resource security problems and consequences. Figure 1 shows the causal relationships within the model: the driving (D) force acts on the system, triggering the pressure (P), which causes changes in the system state (S), which then induce an impact (I) on the system, which in turn prompts various responses (R) to the changes in the state that act on the system as a whole, including the driver (D), the pressure (P), the state (S), and the impact (I). Therefore, the systematic features and the DPSIR model comprehensiveness can effectively study mineral resource security and identify and clarify the relationships between the different units. The second advantage is that the DPSIR model connects the five plates to form a closed single direction loop; consequently, when the equilibrium state is broken, the problem can be identified immediately and the cause targeted. Therefore, this paper selected the relatively comprehensive DPSIR framework to develop the evaluation system.

### 1.5. E-DEMATEL-TODIM Method

When the comprehensive index system is identified, the next step is to choose an appropriate evaluation method and determine the weight of each indicator. Commonly used subjective weight methods have been AHP (Analytic hierarchy process), ANP (Analytic network process), and the DEMATEL (Decision making trial and evaluation laboratory) method. However, the AHP does not consider the connections between the indicators, and the ANP method has consistency problems. However, the DEMATEL method not only calculates the weights but can be used to analyze the causality between the factors. Further to reduce the subjectivity, the entropy method has been widely employed. Therefore, to balance the advantages of these two methods, this paper combined the DEMATEL method with the entropy method (E-DEMATEL) to ensure that the calculated index weights reflected both the subjective and the objective information. After obtaining the comprehensive weights, many methods can be used to assess the energy security performances. For example, Yuan et al. [49] adopted an improved TOPSIS (technique for order preference by similarity to ideal solution) method to assess China’s regional energy security performance, Shah et al. [50] used a composite index approach to measure energy security and conduct an environmental security assessment, and Zhang et al. [13] proposed a fuzzy PROMETHEE (Preference Ranking Organization Method for Enrichment of Evaluations) method to assess the energy security performances within China. While the above methods provide various ways to evaluate energy security, they all have some limitations. For example, when the differences between the years are small, the TOPSIS method is unable to provide a clear explanation, and while the other methods can overcome this drawback, their respective assessment values can only be expressed using crisp numbers. Given the diversity of the qualitative and quantitative evaluation indicator information, the fuzzy TODIM (an acronym in Portuguese for Interactive and Multicriteria Decision Making) method [51,52] was used in this paper. TODIM can measure the degree of dominance for each alternative over other alternatives and then evaluates and ranks the alternatives based on certain features; therefore, it has proven to be a valuable tool for dealing with many comprehensive evaluation problems. Further as TFN (triangular fuzzy number) have been found to be effective in reducing the ambiguity and uncertainty of qualitative indicators, TFN were also applied to the TODIM method and the weighting process.

## 2. Contribution and Objectives

There have been many energy/resource security evaluation models established; however, none have been able to overcome massive information loss and distortion, fully cover the multiple system elements, highlight the relative independence between the indicators, produce unilateral evaluation results, or overview the comprehensive index and reveal the links between the indicators.

Therefore, to deal with these issues, this paper makes several significant improvements. First, different from previous index systems, the DPSIR analysis framework is first combined with a mineral security evaluation, which enriches the sustainability and international energy/resource security indicators and fully reflects the logical relationships between the indicators. Second, because of the scarcity of rare earth related data, hybrid indicators are used and an extended E-DEMATEL-TODIM method integrated with TFN to reduce information loss and avoid individual bias because of the lack of practical experience data.

Therefore, with these considerations in mind, the final objectives of this paper were: (a) to build a rare earth security index system based on the DPSIR model; (b) to assess the rare earth security level in China using the E-DEMATEL-TODIM method; and (c) to investigate the elements influencing China’s rare earth security performance and provide a useful reference for future healthy, sustainable development.

## 3. Methodology

**Figure 2:**
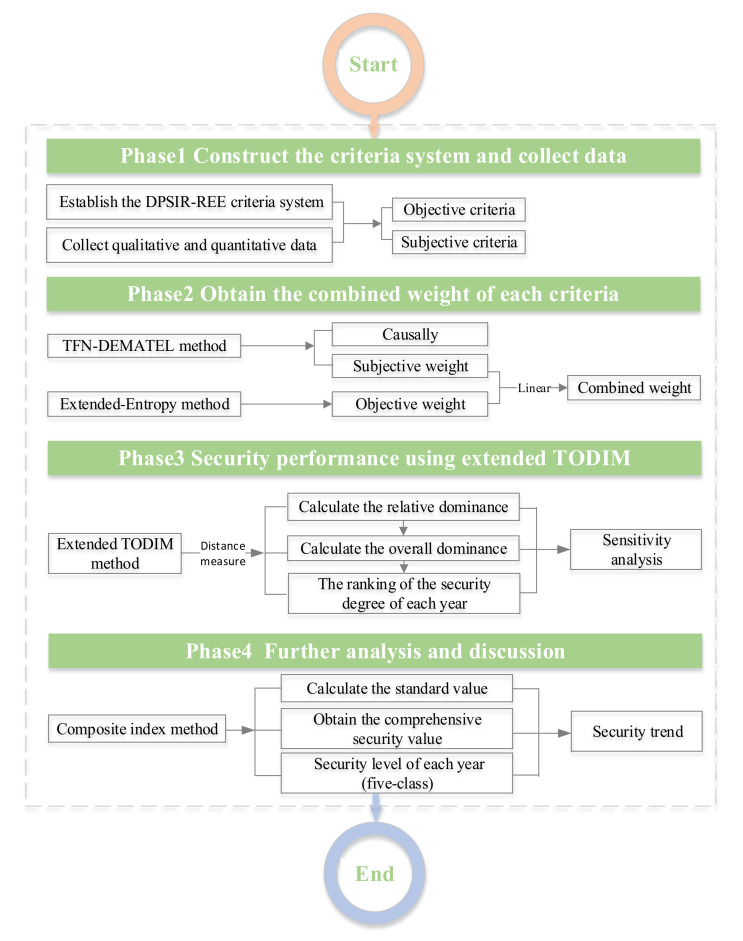
Security performance evaluation framework for Chinese REEs resources.

An extended TODIM method under a fuzzy environment is presented in this section. The framework for the method is shown in Figure 2. This approach simultaneously combined fuzzy sets, linear weights, and the extended TODIM: the linear weight was associated with the TFN-DEMATEL and the extended entropy methods; the fuzzy sets were used to express the uncertainty and the ambiguity of the index; the linear weight method was used to obtain the facto weights; the extended TODIM method was adopted to assess the security degree in each year; and finally, the composite index method was used to calculate the comprehensive security values in each year.

### 3.1. Triangular Fuzzy Number (TFN)

Fuzzy set theory was first introduced by Zadeh [53], after which various types of fuzzy sets were developed in many different fields as TFN were found to be effective in reducing the ambiguity and uncertainty of qualitative indicators.

**Definition 1.**  *If $\tilde{i}=[i^{l},i^{m},i^{u}]=\left\{i\left|0<i^{l}\leq i^{m}\leq i^{u},i,i^{l},i^{m},i^{u}\in R\right.\right\}$ then the $\tilde{i}$ is called a TFN. where, $i^{l},i^{m},i^{u}$ are respectively the lower, median and upper limits of the triangular fuzzy number $\tilde{i}$. The mathematical membership function is expressed as follows:*

(1) $$u_{\tilde{i}}(i)=\left\{\begin{array}{c} i-i^{l}/i^{m}-i^{l},\\ i^{u}-i/i^{u}-i^{m},\\ 0, \end{array}\right.\begin{array}{c} fi^{l}\leq i\leq i^{m}\\ fi^{m}\leq i\leq i^{u}\\ otherwise \end{array}$$

The operational laws for the two TFNs $\left(i_{1}{}^{l},i_{1}{}^{m},i_{1}{}^{u}\right)$ and $\left(i_{2}{}^{l},i_{2}{}^{m},i_{2}{}^{u}\right)$ are defined as:

(2) $$\begin{array}{c} \left(i_{1}{}^{l},i_{1}{}^{m},i_{1}{}^{u}\right)⊕\left(i_{2}{}^{l},i_{2}{}^{m},i_{2}{}^{u}\right)=\left(i_{1}{}^{l}+i_{2}{}^{l},i_{1}{}^{m}+i_{2}{}^{m},i_{1}{}^{u}+i_{2}{}^{u}\right)\\ \left(i_{1}{}^{l},i_{1}{}^{m},i_{1}{}^{u}\right)-\left(i_{2}{}^{l},i_{2}{}^{m},i_{2}{}^{u}\right)=\left(i_{1}{}^{l}-i_{2}{}^{l},i_{1}{}^{m}-i_{2}{}^{m},i_{1}{}^{u}-i_{2}{}^{u}\right)\\ \left(i_{1}{}^{l},i_{1}{}^{m},i_{1}{}^{u}\right)⊗\left(i_{2}{}^{l},i_{2}{}^{m},i_{2}{}^{u}\right)=\left(i_{1}{}^{l}i_{2}{}^{l},i_{1}{}^{m}i_{2}{}^{m},i_{1}{}^{u}i_{2}{}^{u}\right)\\ \frac{i_{1}{}^{l},i_{1}{}^{m},i_{1}{}^{u}}{i_{2}{}^{l},i_{2}{}^{m},i_{2}{}^{u}}=\left(\frac{i_{1}{}^{l}}{i_{2}{}^{l}},\frac{i_{1}{}^{m}}{i_{2}{}^{m}},\frac{i_{1}{}^{u}}{i_{2}{}^{u}}\right)\\ {\left(i_{1}{}^{l},i_{1}{}^{m},i_{1}{}^{u}\right)}^{-1}=\left(\frac{1}{i_{1}{}^{u}},\frac{1}{i_{1}{}^{m}},\frac{1}{i_{1}{}^{l}}\right) \end{array}$$

The distance calculation formula is:

(3) $$D\left({\tilde{i}}_{1},{\tilde{i}}_{2}\right)=\sqrt{\frac{{\left(i_{1}{}^{l}-i_{2}{}^{l}\right)}^{2}+{\left(i_{1}{}^{m}-i_{2}{}^{m}\right)}^{2}+{\left(i_{1}{}^{u}-i_{2}{}^{u}\right)}^{2}}{3}}$$

A comparison of two TFNs is then conducted to rank the results and a method applied to transform the TFNs into corresponding crisp numbers, the formula for which is

(4) $$F(\tilde{i})=\frac{i^{l}+4i^{m}+i^{u}}{6}$$

Therefore, when the two TFNs ${\tilde{i}}_{1}(i_{1}^{l},i_{1}^{m},i_{1}^{u})$ and ${\tilde{i}}_{2}(i_{2}^{l},i_{2}^{m},i_{2}^{u})$ are compared, the following rules are obtained:

(5) $$\left\{\begin{array}{c} {\tilde{i}}_{1}>{\tilde{i}}_{2},\;F\left({\tilde{i}}_{1}\right)>F\left({\tilde{i}}_{2}\right)\\ {\tilde{i}}_{1}={\tilde{i}}_{2},if\;F\left({\tilde{i}}_{1}\right)=F\left({\tilde{i}}_{2}\right)\\ {\tilde{i}}_{1}<{\tilde{i}}_{2},\;F\left({\tilde{i}}_{1}\right)<F\left({\tilde{i}}_{2}\right) \end{array}\right.$$

### 3.2. Triangular Fuzzy Number–Decision Making Trial and Evaluation Laboratory (TFN-DEMATEL)

The DEMATEL methodology, which employs graph theory and matrix tools to analyze the direct and indirect relationships between indicators in a complex system according to type and severity, was originally developed by the Battelle Memorial Institute at the Geneva Research Center [54]. As DEMATEL can also determine the primary and secondary relationships between elements based on a direct influence matrix, it has been widely used in many fields.

As the traditional DEMATEL method is able to characterize evaluative information and can reflect expert judgement uncertainty, in this paper, the expert linguistic variables were transformed into triangular fuzzy number to improve the process, with the TFN—DEMATEL method being as follows:

Step 1. Establish assessment linguistic set.

Fuzzy set theory and triangular fuzzy numbers were applied to quantify the subjective judgements of the expert groups. Then, a five-scale linguistic set was used to describe the degree of influence between the factors [55], as shown in Table 2.

Step 2. Construct the fuzzy direct influence matrix.

The experts described the factors based on the above linguistic sets, with ${\tilde{z}}_{ij}^{k}=(a_{ij}^{k},b_{ij}^{k},c_{ij}^{k})$ representing the TFN; that is, the influence factor i has on factor given by expert k. The triangular fuzzy direct influence matrix ${\tilde{Z}}_{ij}^{k}$ given by each expert was:

(6) $${\tilde{Z}}^{k}=\left[\begin{array}{cccc} 0 & {\bar{z}}_{12}{}^{k} & \cdots & {\bar{z}}_{1j}{}^{k}\\ {\bar{z}}_{21}{}^{k} & 0 & \cdots & {\bar{z}}_{1j}{}^{k}\\ \vdots & \vdots & \vdots & \vdots \\ {\bar{z}}_{i1}{}^{k} & {\bar{z}}_{i2}{}^{k} & \cdots & 0 \end{array}\right]\begin{array}{c} k=1,2,\cdots ,p\\ i=1,2,\cdots ,n\\ j=1,2,\cdots ,n \end{array}$$

Step 3. Calculate the crisp values.

To defuzzify the data, the Converting Fuzzy data into Crisp Scores (CFCS) method was employed because of its simple calculation and small numerical losses, as follows:

First, the fuzzy direct influence matrix was standardized, the processing method for which was as follows:

(7) $$\left\{\begin{array}{l} l_{ij}^{k}=\frac{\left(a_{ij}^{k}-\underset{1\leq k\leq p}{\min}a_{ij}^{k}\right)}{\Delta_{\min}^{\max}}\\ m_{ij}^{k}=\frac{\left(b_{ij}^{k}-\underset{1\leq k\leq p}{\min}b_{ij}^{k}\right)}{\Delta_{\min}^{\max}}\\ r_{ij}^{k}=\frac{\left(c_{ij}^{k}-\underset{1\leq k\leq p}{\min}c_{ij}^{k}\right)}{\Delta_{\min}^{\max}}\\ \Delta_{\min}^{\max}=\underset{1\leq k\leq p}{\max}c_{ij}^{k}-\underset{1\leq k\leq p}{\min}a_{ij}^{k} \end{array}\right.$$

where $l_{ij}^{k}$, $m_{ij}^{k}$, $r_{ij}^{k}$ were the left, middle and right bounds of the normal TFN of matrix ${\tilde{Z}}_{ij}^{k}$, $a_{ij}^{k}$,$b_{ij}^{k}$, $c_{ij}^{k}$, were the original TFNs for the matrix, and $c_{ij}^{k}$ and $a_{ij}^{k}$ were respectively the largest upper bound and the smallest lower bound in each matrix column.

Then, the left and right normal value bounds were calculated, as follows:

(8) $$\left\{\begin{array}{l} u_{ij}^{k}=\frac{m_{ij}^{k}}{\left(1+m_{ij}^{k}-l_{ij}^{k}\right)}\\ n_{ij}^{k}=\frac{r_{ij}^{k}}{\left(1+r_{ij}^{k}-m_{ij}^{k}\right)} \end{array}\right.$$

where $u_{ij}^{k}$ and $n_{ij}^{k}$ were respectively the left and right bounds for the normal TFNs in the matrix.

The standardized crisp values were then calculated, as follows:

(9) $$z_{ij}^{k}=\underset{1\leq k\leq n}{\min}l_{ij}^{k}+\frac{u_{ij}^{k}(1-u_{ij}^{k})+n_{ij}^{k}\times n_{ij}^{k}}{1-u_{ij}^{k}+n_{ij}^{k}}\Delta_{\min}^{\max}$$

where $z_{ij}^{k}$ was the comprehensive normalized value for the influence of factor i obtained from the kth expert on factor j.

Finally, the final crisp values were calculated, as follows:

(10) $$z_{ij}=\frac{1}{n}\sum_{k=1}^{n}z_{ij}^{k}$$

The $\tilde{Z}$ matrix was then converted into a real number type direct influence matrix $\tilde{Z}$ (element $z_{ij}$).

Step 4. Normalize the direct influence matrix Z

.

Matrix was normalized as follows:

(11) D=p⋅Z

(12) $$p=\frac{1}{\underset{1\leq i\leq n}{\max}\left(\sum_{j=1}^{n}z_{ij}\right)}$$

Step 5. Calculate the comprehensive influence matrix.

Matrix D was the standardized direct influence matrix, with the comprehensive influence matrix T being obtained as follows:

(13) $$T=D{\left(1-D\right)}^{-1}$$

Step 6. Construct the TFN-DEMATEL map.

$R_{j}$ and $C_{j}$ were the sum of the rows and columns associated with matrix T, the processing for which was

(14) $$T={\left[t_{ij}\right]}_{n\times n}(i,j=1,2,\cdots ,n)\;R_{i}=\sum_{j=1}^{n}t_{ij}={\left[r_{j}\right]}_{1\times n}$$

(15) $$C_{j}=\sum_{i=1}^{n}t_{ij}={\left[c_{i}\right]}_{n\times 1}$$

where $\left(R_{i}+C_{j}\right)$ was impact of this factor on the whole system and $\left(R_{i}-C_{j}\right)$ was the importance of the factor index; if the index was positive, it meant that it affected other indicators, but if it was negative, it indicated that the factor was being influenced by others.

Step 7. Determine the subjective weights of the criterion.

The subjective weights for each criterion were determined as follows:

(16) $$w^{\prime }{}_{ij}={\left[{\left(r_{j}+c_{i}\right)}^{2}+{\left(r_{j}-c_{i}\right)}^{2}\right]}^{1/2}$$

(17) $$w_{ij}=\frac{w^{\prime }{}_{ij}}{\sum_{j=1}^{n}w^{\prime }{}_{ij}}$$

### 3.3. Entropy Method Based on Mixed Information

The entropy weight method is a data processing method that determines objective weights depending on the size of the index variability, with the information entropy parameter $E_{j}$ indicating the state of the information. Generally speaking, the smaller (larger) the $E_{j}$ of an indicator, the greater (smaller) the degree of indicator value variation, and the more (less) information provided, the greater (smaller) the role it plays in the comprehensive evaluation, and the greater (smaller) the weight. Generally, the entropy weight method is used to calculate real values and objective factors; however, as the indicator system in this paper included both subjective and objective factors, TFN was introduced into the processing, as follows:

Step 1. Construct the original hybrid matrix.

To weight both the qualitative and quantitative indexes, the original matrix included both real numbers and the TFNs; therefore, the hybrid matrix I was:

(18) $$I=\left[\begin{array}{ccccccc} i_{11} & i_{12} & \cdots & {\tilde{i}}_{1r} & {\tilde{i}}_{1,r+1} & \cdots & {\tilde{i}}_{1,n}\\ i_{21} & i_{22} & \cdots & {\tilde{i}}_{2r} & {\tilde{i}}_{2,r+1} & \cdots & {\tilde{i}}_{2,n}\\ \vdots & \vdots & \ddots & \vdots & \vdots & \ddots & \vdots \\ i_{m1} & i_{m2} & \cdots & {\tilde{i}}_{mr} & {\tilde{i}}_{m,r+1} & \cdots & {\tilde{i}}_{m,n} \end{array}\right]$$

where $i_{ij}$, a real number, indicated the value of the quantitative index elements, and $i_{ij}$ indicated the TFNs.

Step 2. Standardize the mixed indicators.

Different criteria need to be normalized using different methods. The initial hybrid was standardized using Equations (19)–(22), as follows.

For the real numbers for the positive factors, the standardized value was calculated as follows;

(19) $$x_{ij}=\frac{i_{ij}-\underset{j}{\min}i_{ij}}{\underset{j}{\max}i_{ij}-\underset{j}{\min}i_{ij}}$$

For the real numbers for the negative factors, the standardized value was calculated as follows;

(20) $$x_{ij}=\frac{\underset{j}{\max}i_{ij}-i_{ij}}{\underset{j}{\max}i_{ij}-\underset{j}{\min}i_{ij}}$$

For the TFNs for the positive factors, the standardized value was calculated as follows;

(21) $${\tilde{x}}_{i,j}=({\tilde{x}}_{i,j}^{l},{\tilde{x}}_{i,j}^{m},{\tilde{x}}_{i,j}^{n})=\left(\frac{{\tilde{i}}_{i,j}^{l}}{\underset{i}{\max}({\tilde{i}}_{ij})},\frac{{\tilde{i}}_{i,j}^{m}}{\underset{i}{\max}({\tilde{i}}_{ij})},\frac{{\tilde{i}}_{i,j}^{u}}{\underset{i}{\max}({\tilde{i}}_{ij})}\right)$$

For the TFNs for the negative factors, the standardized value was calculated as follows;

(22) $${\tilde{x}}_{i,j}=({\tilde{x}}_{i,j}^{l},{\tilde{x}}_{i,j}^{m},{\tilde{x}}_{i,j}^{n})=\left(\frac{\underset{i}{\min}({\tilde{i}}_{ij})}{{\tilde{i}}_{i,j}^{l}},\frac{\underset{i}{\min}({\tilde{i}}_{ij})}{{\tilde{i}}_{i,j}^{m}},\frac{\underset{i}{\min}({\tilde{i}}_{ij})}{{\tilde{i}}_{i,j}^{u}}\right)$$

Finally, the standardized matrix for the standardized values was developed:

(23) $$X=\left[\begin{array}{ccccccc} x_{11} & x_{12} & \cdots & {\tilde{x}}_{1r} & {\tilde{x}}_{1,r+1} & \cdots & {\tilde{x}}_{1,j}\\ x_{21} & x_{22} & \cdots & {\tilde{x}}_{2r} & {\tilde{x}}_{2,r+1} & \cdots & {\tilde{x}}_{2,j}\\ \vdots & \vdots & \ddots & \vdots & \vdots & \ddots & \vdots \\ x_{mn} & x_{i2} & \cdots & {\tilde{x}}_{ir} & {\tilde{x}}_{i,r+1} & \cdots & {\tilde{x}}_{i,j} \end{array}\right]$$

Step 3. Calculate the information entropy for each criterion.

Because of the hybrid criteria included in matrix X, an extended entropy method was adopted for the mixed information calculation, for which the distance between the elements was used to express the information entropy, the formula for which was

(24) $$h_{j}=-\frac{1}{\ln m}\sum_{i=1}^{m}\left[\frac{d\left(q_{ij},{\bar{q}}_{j}\right)}{\sum_{i=1}^{m}d\left(q_{ij},{\bar{q}}_{j}\right)}\ln\left(\frac{d\left(q_{ij},{\bar{q}}_{j}\right)}{\sum_{i=1}^{m}d\left(q_{ij},{\bar{q}}_{j}\right)}\right)\right]$$

(25) $$\begin{array}{l} {\bar{q}}_{j}=(q_{1j}⊕\cdots ⊕q_{ij}⊕q_{imj})/m\\ =\left\{\begin{array}{l} \frac{1}{m}\sum_{i=1}^{m}x_{ij},\\ \left(\frac{1}{m}\sum_{i=1}^{m}{\tilde{x}}_{ij}^{l},\frac{1}{m}\sum_{i=1}^{m}{\tilde{x}}_{ij}^{m},\frac{1}{m}\sum_{i=1}^{m}{\tilde{x}}_{ij}^{u}\right), \end{array}\right. \end{array}$$

where $q_{ij}$ was the element of matrix X, and $d\left(q_{ij},{\bar{q}}_{j}\right)$ was the distance.

Step 4. Calculate the objective weight of each factor.

The final step in calculating the objective weight was as follows:

(26) $$w_{qj}=\frac{1-h_{j}}{\sum_{j=1}^{n}1-h_{j}}$$

Therefore, using the above processes, the final weights for both the quantitative and qualitative criteria were obtained. To combine the advantages of the two methods, they were then aggregated using a linear weighting method, as follows:

(27) $$w_{j}=\alpha w_{ij}+\beta w_{qj}$$

where α+β=1 and α,β≥0. The value of these two parameters can be adjusted by decision makers based on their preference; however, as the two weights were considered equally important in this paper, α=β=0.5.

### 3.4. Extended TODIM Method with Mixed Value

As in prospect theory, the TODIM method considers psychological behavior and uses the dominance degree of one alternative over another to rank objects. The traditional TODIM method concentrates on real number calculations and keeps the loss attenuation factor θ stable; however, as this paper introduced TFNs, the θ was changed, as follows:

Step 1. Obtain the normalized matrix and calculate the relative index weights.

It was assumed that matrix $Q={\left[q_{ij}\right]}_{m\times n}$ included a set of alternatives $A=\left(A_{1},A_{2},\cdots A_{m}\right)$ and the criteria set $C=\left(C_{1},C_{2},\cdots ,C_{n}\right)$. Equations (19)–(22): the standardization of the objective and subjective criteria have been explained, with the data from $Q={\left[q_{ij}\right]}_{m\times n}$ being found to have met the standardization requirements. Then, the relative weight for $C_{j}$ was calculated:

(28) $$w_{jt}=\frac{w_{j}}{w_{t}}$$

where $w_{t}=\max\left\{w_{j}\left|j\in n\right.\right\}$.

Step 2. Compute the relative dominance under one factor.

Different from the traditional TODIM, the distance was used to express the deviations between the two criteria, with the different numbers being handled as previously outlined. The relative dominance was determined as follows:

(29) $$\phi_{j}\left(A_{i},A_{k}\right)=\left\{\begin{array}{c} \sqrt{\frac{w_{jt}}{\sum_{j=1}^{m}w_{jt}}d\left(q_{ij},q_{kj}\right)}\\ 0\\ -\frac{1}{\theta }\sqrt{\frac{\sum_{j=1}^{m}w_{jt}}{w_{jt}}d\left(q_{ij},q_{kj}\right)} \end{array}\begin{array}{c} q_{ij}>q_{kj}\\ \begin{array}{c} q_{ij}=q_{kj} \end{array}\\ q_{ij}<q_{kj} \end{array}\right.$$

where θ was the recession coefficient, that is, the risk and loss avoidance psychology of the decision makers; the smaller the θ, the higher the psychological level of decision-maker’s loss avoidance.

Step 3. Calculate the overall dominance involving all criteria.

The overall dominance of each scheme was then calculated:

(30) $$\delta \left(A_{i},A_{k}\right)=\sum_{j=1}^{n}\phi_{j}\left(A_{i},A_{k}\right)$$

Step 4. Determine the global prospect value and rank the alternatives.

Finally, the comprehensive ranking value $\zeta_{i}$ was determined, as follows:

(31) $$\zeta_{i}=\frac{\sum_{k=1}^{m}\delta \left(A_{i},A_{k}\right)-\underset{i}{\min}\sum_{k=1}^{m}\delta \left(A_{i},A_{k}\right)}{\underset{i}{\max}\sum_{k=1}^{m}\delta \left(A_{i},A_{k}\right)-\underset{i}{\min}\sum_{k=1}^{m}\delta \left(A_{i},A_{k}\right)}$$

### 3.5. Composite Index Method

The composite index method was used to calculate the comprehensive value of the security evaluation, and based on the results, the security evaluation was divided into a five-level evaluation. The evaluation rating standards are shown in Table 1, the composite index for which was calculated as follows:

(32) $$E=X^{\prime }\times w_{j}$$

where $X^{\prime }$ was the normalized matrix calculated from matrix $X^{\prime }$, for which the subjective indicators were processed using Equation (4).

## 4. Security Assessment of Chinese REEs Resources

### 4.1. Problem Description

An evaluation framework for Chinese REEs security from 2006 to 2015 was constructed and comprehensive performance analyses performed, after which management advice on Chinese REEs security improvements was given. The framework is shown in Figure 2, and the specific process is summarized as follows:

Phase 1. Construct the criteria index and collect the data.

The criteria were divided into five parts, which included 3 qualitative indicators and 27 quantitative indicators based on the DPSIR model, with the objective data obtained from research reports and websites, and the subjective data obtained from expert surveys and questionnaires. To facilitate the calculations, the language expressions collected from the experts were transformed into TFNs and combined with the real numbers in the decision-making matrix.

Phase 2. Obtain the combined weight.

First, the subjective weights were calculated using the TFN-DEMATEL method, which also elucidated the causal relationships between the indicators. Then the objective weights were calculated using the entropy method, with the entropy information being expressed using the distance measure to adjust the hybrid matrix.

Phase 3. Assess the security performance using the proposed extended-TODIM method.

The TODIM method was adopted to rank the security performances in each year, with the recession coefficient being set to 1 for all the indicators. Then, to verify the stability and the adaptability of the TODIM method, the parameter was set from 0.1 to 2.25 to assess the changes in the ranking results.

Phase 4. Further analysis and discussion.

Based on the ranking results, the composite index method was applied to calculate the comprehensive security value in each year and classify the composite scores into five-classes. Lastly, a factor analysis was employed for the scientific assessment.

### 4.2. Determination of the Evaluation Indexes and Data Sources

**Figure 3:**
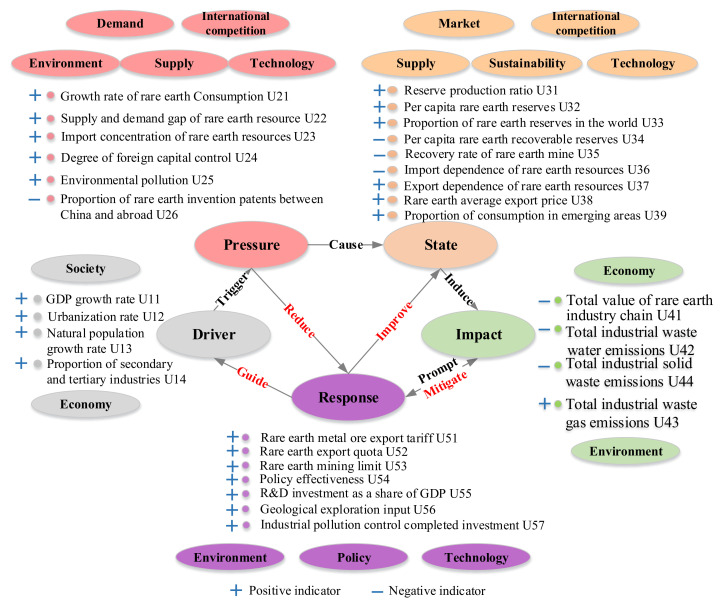
Index system diagram.

Thirty indicators were adopted for the REEs security performance criteria system construction, which included five main indicator sets: driver indicators, pressure indicators, state indicators, influence indicators, and response indicators, as shown in Figure 3.

#### 4.2.1. Driver Indicators

Resource security is related to the security of the combined resource, environment, and socio-economic systems, with the driving force being the potential force needed to affect change in the REEs security situation. The resource security system is impacted when there is rapid population growth; extensive regional economic, urban, industrial, and technological developments; and corresponding lifestyle, resource consumption, and production changes. As rare earth resource consumption growth is directly influenced by the gross domestic product (GDP) and industrial growth rate and rapid population and urban growth, the research and development (R&D) investment proportion in GDP represents the technological investment.

#### 4.2.2. Pressure Indicators

The rapid development of China’s economy and society has led to a significant increase in the demand for rare earth resources, which has increased demand and supply pressure, which in turn has increased the resource pressure. If the in-country resource is unable to supply national needs, it is necessary to seek the excess demand overseas; however, excessive dependence on imports can put pressure on resource security. The security of the industry is also affected when the rare earth demand increases due to changes in science and technology; therefore, from an industrial structure perspective, the degree of control over foreign investment reflects the controllability of the rare earth industry in China. However, while the resources needed for survival and development are being extracted from the natural system, waste from the energy and system environment results in environmental pressure.

#### 4.2.3. State Indicators

The resource security state is the intuitive representation under pressure, which mainly refers to indicator stability: resource supply, resource availability, international trade, and innovation; therefore, five indicators, reserve production ratio, per capita rare earth reserves, proportion of rare earth reserves in the world, per capita rare earth recoverable reserves, and rare earth mine recovery rates, and three international trade state indexes, import dependence on rare earth resource, export dependence on rare earth resource, and rare earth average export price, were selected, with the innovation state being determined based on the consumption proportion in emerging areas.

#### 4.2.4. Impact Indicators

The rare earth resource impact refers to the overall system after all pressures and state changes have been considered. The examination of the impact of the pressures on security is related to the system status quo and the influence of the resource and is the basis for the formulation of correction strategies. Four indexes were applied: the total value of the rare earth industry chain, the total industrial waste water emissions, the total industrial waste gas emissions, and the total industrial solid waste emissions, for which the indicators were selected based on rare earth industry economic development and environmental aspects, which are the key rare earth security system output factors. Therefore, the total value for the rare earth industry chain was selected to represent industry development, and total industrial wastewater emissions, total industrial waste gas emissions, and total industrial solid waste emissions were selected to represent the current environmental impacts.

#### 4.2.5. Response Indicators

The rare earth metal ore export tariff, the rare earth export quota, the rare earth mining limit, policy effectiveness, R&D investment as a share of GDP, geological exploration input, and completed industrial pollution control investment were selected as the response indexes to reflect the response degree of national policies, industry and society to the stress and status indicators. The rare earth metal ore export tariff, the rare earth export quota, the rare earth mining limit, and the policy effectiveness together reflected the policy response, the R&D investment as a share of GDP reflected the industry innovation, and the geological exploration input and completed industrial pollution control investment reflected the resource utilization and environmental protection.

Therefore, the 27 quantitative indicators and the 3 qualitative indicators (shown in Figure 3) were combined to construct the rare earth resource security assessment system. The qualitative indexes were degree of foreign capital control, environmental pollution, and policy effectiveness. The degree of foreign capital control referred to the controllability in the country and included the same proportions of industry technological control and resource control, with the higher the final evaluation value, the greater the pressure. The environmental pollution index measured the environmental damage and pollution caused by rare earth production and had a subjective scoring method; the pollution equally included water, land, air, and vegetation pollution at 25% each, with the higher the final evaluation values, the greater the pressure. The policy effectiveness indicator determined the degree to which the issued and implemented government policies affected the rare earth industry security and health, with the higher the final evaluation value, the greater the effectiveness. The quantitative data used in this paper were extracted from the 2007–2015 China Statistical Yearbooks, the 2007–2016 China Industrial Statistics Yearbooks, the 2007–2016 China Environmental Statistics Yearbooks, and the 2007–2016 China Nonferrous Metal Industry Yearbooks. Data were also obtained from the United States Geological Survey (USGS), the UN Comrade Database, the China Rare Earth website, and the Ministry of Commerce website of the People’s Republic of China.

### 4.3. Implement the Proposed Extended TODIM Method

#### 4.3.1. Obtain the Combined Weight

After collecting the values for each of the corresponding indicators, the weight for each criterion was determined, which was done in three stages.

First, the subjective weights were determined. The experts evaluated the indicators using the linguistic variables shown in Table 2 to assess the degree of influence between each criterion, after which the E-DEMATEL method was used to determine the subjective weights for each criterion.

**Figure 4:**
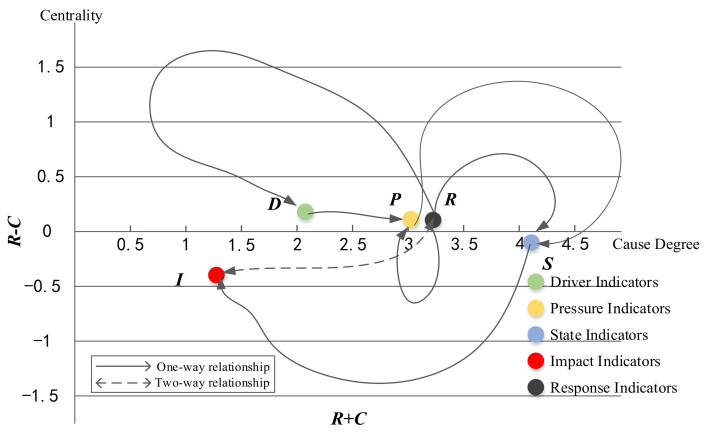
Causal relationships between the criteria.

As stated, the DEMATEL method can also be used to explore the causal relationships between the indicators. As shown in Figure 4, the ordinate was expressed using the cause degree, and the abscissa was expressed using centrality, and the graph also shows the relationships between DPSIR model factors, from which it can be seen that the driver criteria, pressure criteria, and response criteria located above the *X*-axis were the cause indicators, and the impact and state criteria were the result criteria. Therefore, the graph results intuitively verified the relationships in the DPSIR model. The driver factor, as a potential driving force, directly puts pressure on resource use, and the pressure indicator reflects the direct cause for the current resource status change. Then, with a change in the system state, a certain impact is generated; therefore, to eliminate or reduce the impact, certain response measures must be taken to compensate for the effects, restore the state, reduce the stress, and improve the driving power.

The second stage involved calculating the objective weights. Based on the original hybrid matrix, a distance measure method was used to obtain the information entropy, after which Equations (24) and (27) were applied to obtain the final objective weights.

**Figure 5:**
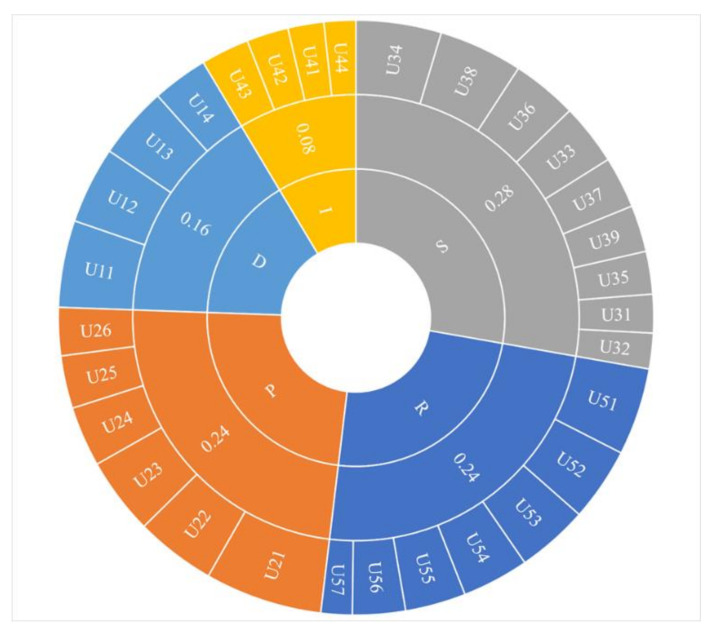
Weights for the criteria on all levels.

Then using Equation (27), the subjective and objective weights were fused to obtain the comprehensive weights for which = 0.5, with the final combined weights for each indicator being shown in Table 3. As the table does not show the target layer weights, to intuitively show the indicator weights on all levels, a corresponding pie chart was drawn up (Figure 5).

#### 4.3.2. Security Performance Evaluation in Each Year

Therefore, by applying the extended-TODIM method, the comprehensive ranked values for the REEs security performance in China from 2006 to 2015 were obtained. Based on the several calculation results, the security performance final order in each year was determined to be as follows: 2012 > 2013 > 2014 > 2011 > 2010 > 2015 > 2007 > 2009 > 2008 > 2006: with the concrete results shown in Table 4.

#### 4.3.3. Sensitivity Analysis

**Figure 6:**
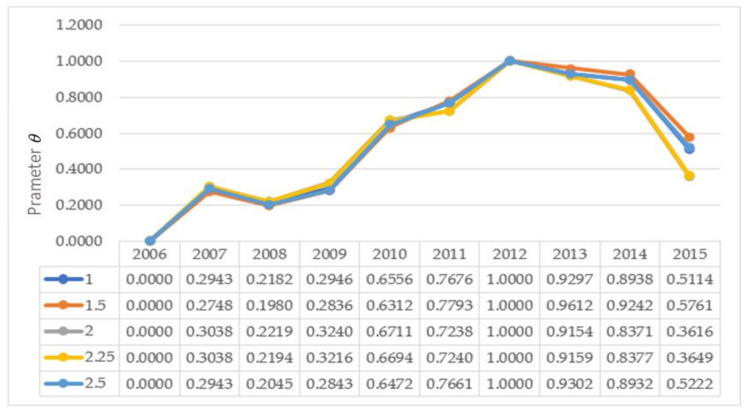
Impact of parameter θ on the decision results.

There was a loss attenuation coefficient θ (θ > 0) included in the formula to calculate the overall dominance between the years, which reflected the decision maker’s sensitivities to the losses; that is, as the value for θ showed the risk aversion attitude of the decision maker, the smaller the θ, the higher the degree risk aversion. Therefore, under different risk aversion attitudes, the security assessment state would be different each year. In existing studies, the two values θ=1 and θ=2.5 have been adopted the most. The order trends for the alternatives for the different θ values are given in Figure 6.

It can be seen that the ranking of the alternatives remained unchanged when θ is equal to 1, 1.5, 2, 2.25 and 2.5, which indicated that although the value of the attenuation factor for the losses changed, the security level ordering did not change each year, with 2013 remaining the best alternative.

### 4.4. Further Analysis and Discussion

**Figure 7:**
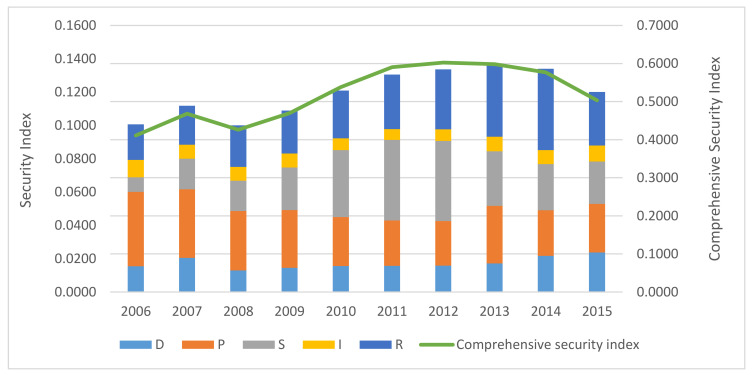
Time-series graph for the coordinated security of the five subsystems from 2006–2015.

With the weight of each indicator known, to finally obtain the security index values for the various levels, the composite index was adapted by multiplying each of the standardized indicators by their respective weights, for which the results are shown in Figure 7.

Comparatively, the final rankings for the comprehensive security index were consistent with the TODIM method ranking. Figure 7 also shows that the change trends of the response elements in the five security evaluation system elements were similar to the changes in the comprehensive security value, which indicated that the security performances were largely driven by the response elements.

As the calculated comprehensive index value cannot determine the “good” and “bad”, to establish the conceptual correlations between the assessment levels and the standardized assessment values, it was necessary to convert the standardized indicator values and the comprehensive index values into grade values. Therefore, the weights for each subsystem were selected as the optimal values, and the comprehensive evaluation values were then calculated, the results of which are shown in Table 5.

The safe standard was then divided into five-class assessment levels: safest, safer, safe, unsafe, and dangerous, with the associated value changes being 90% or more, 75% to 90%, 60% to 45%, and 45% or less. The evaluation rating standard table is shown in Table 6, and the final comprehensive security evaluation results classifications are shown in Table 7.

## 5. Results

### 5.1. Analysis of China’s Comprehensive Rare Earth Security Level Evaluation Results

It was found that China’s rare earth resource security evaluation in the 10-year period from 2006 to 2015 was not high, generally had an upward trend until 2012, and then slightly declined.

The rare earth resource security evaluation from 2006 to 2015 had three main stages. The first stage was from 2006 to 2010, at which time the overall security was in a dangerous or unsafe state but was steadily improving. In 2006, the “Rare Earth Industrial Industry Development Policy” and the “Rare Earth Industry Medium- and Long-Term Development Plan” were issued by the relevant departments, the export tariff scope widened, and exports restricted and strengthened. In the following years, the government further controlled production capacity and no new mining licenses were issued. In 2010, government efforts were focused on shutting down the illegal rare earth resource mining operations, which achieved good results. Overall, therefore, the rare earth production integration and mining quotas had positive effects that both reduced the rare earth resource consumption rate in China and prevented excessive (and illegal) exploitation. However, there were problems in the formulation and implementation of the relevant policies, which affected rare earth industry development and international competitiveness and led to certain rare earth resource security problems.

The second stage was from 2011 to 2013, at which time the rare earth resource security was in a safe state. From 2011, the Chinese government issued several policies to fully regulate rare earth industry development, which included promoting rare earth company integration, formulating emissions standards for rare earth pollution, comprehensively improving the security of rare earth resources in China, and ensuring the healthy and sustainable development of rare earth resources. The results indicated that while these measures had some success, more measures were needed to protect rare earth resource security.

The last stage was from 2014 to 2015, time at which the comprehensive security value declined to an unsafe state compared to the second stage. In 2014, the World Trade Organization (WTO) expert group ruled that China’s rare earth products export management was in violation of regulations, and in 2015, the tariff and export quotas on China’s rare earth exports were cancelled, meaning that other countries’ policy barriers to China’s rare earth resources disappeared. Although this measure stimulated growth in the export volume of individual products, it caused the volume and prices in the rare earth industry in 2015 to fall; therefore, the domestic and international market conditions were not optimistic and the overall losses in the rare earth industry were serious. To completely change the state of the industry, supply-side reform and joint efforts by governments and enterprises were needed.

### 5.2. Subsystem Factor Measurement Results

**Figure 8:**
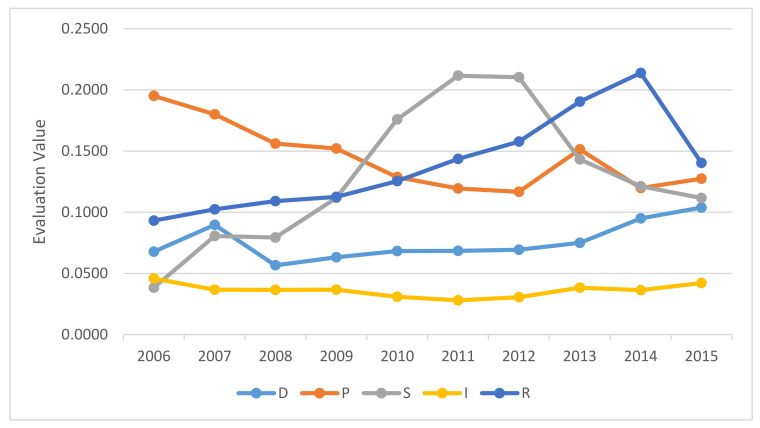
Evaluation values for the security in the five rare earth subsystems from 2006–2015.

To explore the key elements affecting rare earth resource security, the rare earth resource subsystem statuses and the effects of the 30 evaluation indicators on system security were examined using the DPSIR model. Figure 8 shows the trends in each subsystem.

#### 5.2.1. “D” System Analysis

The assessment of the driving subsystem, which was mainly focused on the socio-economic drivers, indicated that the impact effects of each of the indicators were relatively balanced. In the economic force system, however, there were relatively large influences from GDP growth and the proportion of secondary and tertiary industries. Therefore, these two indexes played a crucial role in rare earth industry development, with the tertiary industry in particular stimulating the development of other industries and also promoting emerging growth points. The urbanization rate and the natural population growth rate were also important drivers for rare earth resource security.

#### 5.2.2. “P” System Analysis

The pressure system was associated with resource pressure, trade pressure, technological pressure, and industrial structure pressure, with the rare earth consumption growth rate, the resource supply and demand gap, and the degree of foreign capital control being the main factors putting pressure on resource security. As the rare earth consumption growth rate and the supply and demand resource gap reflect the balance in the use of rare earth, these are conducive to the reasonable allocation of this type of advantageous resources. However, as China’s economy continues to develop, China’s rare earth resource demand increases, and even though China is relatively self-sufficient, China’s rare earth reserves and supply have been poorly coordinated. In particular, as there has been extreme over-exploitation, there needs to be a greater focus on the sustainable development of China’s rare earth resources. Further, even though China began to ban foreign investors from establishing rare earth mining enterprises and restricted foreign investors to investments in related projects and deep processing, there needs to be more focus on corporate behavior within the industry.

#### 5.2.3. “S” System Analysis

The rare earth resource state reflects the state of the system under all pressures. The results indicated that the state index was generally rising but declined in 2012. The weights results showed that the per capita rare earth recoverable reserves and the rare earth average export price played an important role in resource security. Because of the long-term oversupply and the disorderly market supervision, China’s export prices were low, but the mining costs were increasing, which posed a significant threat to the security of the rare earth resources. Therefore, China issued a series of related policies, which included measures to restrict rare-earth mining licenses, to increase export duties, and reduce export quotas. The rare earth mine recovery rate and rare earth advanced application technology competitiveness were also important factors that influenced the rare earth resource security balance.

#### 5.2.4. “I” System Analysis

The impact subsystem indicated the impacts of the changes in the rare earth resource state on the environment and the value of the rare earth industry. The comprehensive index results showed that the impact index decreased in the ten years from 2006 to 2015 and was generally in a dangerous state; there were little obvious rare earth industry value changes, and the environmental state subsystem was deteriorating.

#### 5.2.5. “R” System Analysis

From the five subsystems that made up the rare earth resource security, the change trends in the response system were similar to the comprehensive security value, which indicated that the changes in the security value were largely driven by the response system, with the response index also reaching an inflection point in 2015. The comprehensive security value analysis also reflected the WTO case loss in 2015, at which time the rare earth tariff cancellations removed the national policy barriers and reduced the security index. Although China had liberalized its rare earth export tariffs and lost its tariff advantage, the response could have been improved using other means. The indicators with relatively larger weights in the response system were policy effectiveness, tariffs, and research investment, indicating that measures such as strengthening scientific research investment in rare earth deep processing, increasing prices, improving policy effectiveness, and strengthening supervision could be effective.

To sustainably maintain the rare earth resource security system, key indicators and responses need to be effectively controlled. The quantitative analysis of China’s rare earth resource security based on the DPSIR model found that it was driven by the socio-economic factors and that the resource and industrial structural pressures were increasing, which had resulted in an unbalanced rare earth price and over-exploitation. Further, the increased resource consumption and the associated pollutant emissions led to increased environmental pollution. With these security system problems, responses need to be taken to reduce the pressures, improve the state, effectively mitigate the impacts, and guide the driver, which were resolved based mainly on policy, technology, and environmental protection changes; therefore, response was found to play an important controlling role in the rare earth security system.

## 6. Conclusions

This research constructed an index system to evaluate China’s rare earth resource security level based on a DPSIR model. The DEMATEL method and an extended-entropy weight method were adapted to determine the weights for each index, which included 27 quantitative indicators and 3 qualitative indicators. The causality analysis using the DEMATEL method further confirmed the logical relationships between the DPSIR subsystems and also demonstrated the applicability of DPSIR model in measuring the rare earth resource security level. Then, an extended TODIM method was used to rank the rare earth security levels from 2006 to 2015, with the associated sensitivity analyses verifying the effectiveness of the method. Finally, the results of the comprehensive index method comparatively indicated the consistency of the TODIM method. The final index divided the security level into five levels, with the analysis finding that while China’s rare earth resource security was still not high, the index was generally rising; however, it was only in the basic safe range from 2011 to 2013.

To further analyze the reasons, separate analyses were performed on the five subsystems. It was found that the change trends in the response subsystem were generally consistent with the comprehensive security trends, indicating that the comprehensive security was generally driven by the response system. Socio-economic factors, increasing supply and demand pressures, and severe environmental pollution had led to lower export prices and reduced sustainability. Therefore, based on the above analysis and existing policies, the following policy recommendation are given:

1. Strengthen the management of the development and utilization of China’s rare earth resources

China needs to better activate its ban on illegal rare earth mining enterprises, strictly control rare earth mining, and clamp down on disorderly mining behavior. The government needs to accelerate its establishment of a rare earth resource strategic reserve mechanism, improve its reserve plans, increase the legislative management of rare earth reserves, and implement a strict supervision and inspection system. By improving rare earth resource mining and reserve management systems and mechanisms, the security of rare earth resources could be guaranteed, and a raw materials foundation provided for the future use of China’s rare earth advantages to enhance competitiveness.

2. Enhance rare earth high-tech applications

China’s rare earth resources have been oversupplied for some time. The cancellation of the export quotas and tariffs in 2015 challenged China’s rare earth export prices and rare earth industry security. However, it is possible to increase prices by developing new technologies in emerging fields that require rare earth resources. To do this, however, requires increased independent rare earth research and development, which in turn requires the actioning of national policies. As rare earth is currently listed as a national key supported resource, by combining government funding with the collective wisdom of the industry and scientific research institutions, efforts can be focused on rare earth deep processing developments and new materials application technologies, and by supporting the introduction of foreign rare earth technologies, research and development could be saved and research efficiency improved.

3. Strengthen the “three waste” management of the rare earth industry

The environmental pollution damage caused by rare earth over-exploitation has caused widespread concern. To reduce and even eliminate the environmental damage caused by rare earth industry development, it is necessary to reduce waste at the source by vigorously promoting clean processes, transforming product production technologies and improving industrial technologies. Second, during the production process, waste could be fully utilized; that is, useful substances could be recovered and purified to make other products. Finally, timely wastewater purification and treatment from rare earth production could achieve multiple utilization rounds.

## Figures and Tables

**Table 1:** Representative definitions for energy/mineral resource security.

Sources	Energy/Mineral Resource Security Definitions	Aspects
International Energy Agency [15]	“The physical availability of supplies to satisfy demand at a given price.” “The uninterrupted availability of energy resources at an affordable price.”	Supply/Demand/Price
Shen et al. [16]	“A country or region can control and acquire mineral resources stably, timely and continuously.”	Control/Acquisition
Intharak [17]	“The ability of an economy to guarantee the availability of the energy resource supply in a sustainable and timely manner with the energy price being at a level that will not adversely affect the economic performance of the economy.”	Supply/Price/Effectiveness
Deng [18]	“A country can supply the required mineral resource continuously, stably and in sufficient quantities at a specified time and place at a reasonable price and manner.”	Supply/Price
Leung [19]	“To assure adequate, reliable supplies of energy at reasonable prices and in ways that do not jeopardize major national values and objectives.”	Supply/Price/Society
Martchamadol and Kuma [20]	“The energy security of developing countries refers to “enough energy supply (quantity and quality) to meet all requirements at all times for all citizens at affordable and stable prices, and it also leads to sustainable economic performance and poverty alleviation, and a better quality of life without harming the environment.”	Supply/Price/Society/Environment
Wu [21]	Energy security includes four dimensions: economic security, geopolitical security, environmental security, and military and national security.	Economy/Geopolitical/Environment/Military
Nelwan et al. [22]	7D concepts, including availability, infrastructure, energy prices, social impact, environment, governance, energy efficiency.	Supply/Technology/Price/Society/Environment/Supervision/Effectiveness

**Table 2:** Fuzzy ratings for the linguistic terms.

Linguistic Variables	Corresponding TFNs
no influence (N)	(0, 0.1, 0.3)
very low influence (VL)	(0.1, 0.3, 0.5)
low influence (L)	(0.3, 0.5, 0.7)
high influence (H)	(0.5, 0.7, 0.9)
very high influence (VH)	(0.7, 0.9, 1.0)

**Table 3:** Summary of the criteria weights.

Evaluation System	Indicators	Objective Weights	Subjective Weights	Combined Weights
Driver(D)	U11	0.0311	0.0633	0.0472
	U12	0.0498	0.0288	0.0393
	U13	0.0370	0.0235	0.0302
	U14	0.0554	0.0283	0.0418
Pressure(P)	U21	0.0684	0.0589	0.0637
	U22	0.0441	0.0431	0.0436
	U23	0.0280	0.0293	0.0286
	U24	0.0170	0.0666	0.0418
	U25	0.0339	0.0316	0.0327
	U26	0.0359	0.0164	0.0262
State(S)	U31	0.0234	0.0185	0.0210
	U32	0.0322	0.0066	0.0194
	U33	0.0358	0.0309	0.0333
	U34	0.0371	0.0554	0.0463
	U35	0.0301	0.0163	0.0232
	U36	0.0204	0.0508	0.0356
	U37	0.0242	0.0319	0.0280
	U38	0.0266	0.0646	0.0456
	U39	0.0282	0.0224	0.0253
Impact(I)	U41	0.0304	0.0042	0.0173
	U42	0.0325	0.0129	0.0227
	U43	0.0295	0.0231	0.0263
	U44	0.0295	0.0095	0.0195
Response(R)	U51	0.0254	0.0545	0.0400
	U52	0.0268	0.0391	0.0329
	U53	0.0293	0.0433	0.0363
	U54	0.0565	0.0384	0.0475
	U55	0.0211	0.0129	0.0170
	U56	0.0307	0.0472	0.0389
	U57	0.0296	0.0278	0.0287

**Table 4:** Final values and ordering.

Alternatives	Summation	Standardization	Ranking
2006	−560.1777	0.0000	10
2007	−476.4108	0.2943	8
2008	−498.0707	0.2182	9
2009	−476.3160	0.2946	7
2010	−373.5407	0.6556	5
2011	−341.6629	0.7676	4
2012	−275.5153	1.0000	1
2013	−295.5187	0.9297	2
2014	−305.7402	0.8938	3
2015	−414.6125	0.5114	6

**Table 5:** Weights for each subsystem.

Subsystem	Drive	Pressure	State	Impact	Response	Comprehensive
The optimal value	0.1586	0.2366	0.2776	0.0858	0.2413	0.2238

**Table 6:** Rare earth security performance level evaluation scale.

Security Level	Drive	Pressure	State	Impact	Response	Comprehensive
safest	[0.1427,0.1586)	[0.2129,0.2366)	[0.2498,0.2776)	[0.0772,0.0858)	[0.2172,0.2413)	[0.2014,0.2238)
safer	[0.1190,0.1427)	[0.1775,0.2129)	[0.2082,0.2498)	[0.0644,0.0772)	[0.1810,0.2172)	[0.1678,0.2014)
safe	[0.0952,0.1190)	[0.1420,0.1775)	[0.1666,0.2082)	[0.0515,0.0644)	[0.1448,0.1810)	[0.1343,0.1678)
unsafe	[0.0714,0.0952)	[0.1065,0.1420)	[0.1249,0.1666)	[0.0386,0.0515)	[0.1086,0.1448)	[0.1007,0.1343)
danger	[0.0159,0.0714)	[0.0237,0.1065)	[0.0278,0.1249)	[0.0086,0.0386)	[0.0241,0.1086)	[0.0224,0.1007)

**Table 7:** Comprehensive rare earth resource evaluation security level from 2006–2015.

**Year**	2006	2007	2008	2009	2010	2011	2012	2013	2014	2015
**Grade**	danger	unsafe	danger	unsafe	unsafe	safe	safe	safe	unsafe	unsafe
